# Predicting ICU transfer for high-risk patients upon medical admission via the medical intensive care prediction score (MICAPS)

**DOI:** 10.1186/s12873-025-01448-w

**Published:** 2026-01-08

**Authors:** Muhammad Zahid, Fateen Ata, Adeel Ahmad Khan, Prem Chandra, Rajvir Singh, Abdelnaser Y. Awad Elzouki, Dabia Hamad S. H. Al. Mohanadi, Ahmed Ali A. A. Al-Mohammed

**Affiliations:** 1 Department of Internal Medicine, Hamad General Hospital, Hamad Medical Corporation, Doha, 3050 Qatar; 2 College of Medicine, Qatar University, Doha, Qatar; 3 Weill Cornell Medicine, Doha, Qatar; 4 Department of Internal Medicine, Cleveland Clinic Foundation, Cleveland, OH USA; 5 Department of Medicine, Cleveland Clinic Akron General Hospital, Akron, OH USA; 6 Medical Research Center, Academic Health Systems, Hamad Medical Corporation, Doha, Qatar; 7 Cardiology Research Center, Heart Hospital, Hamad Medical Corporation, Doha, Qatar

**Keywords:** Emergency departments, Intensive care, Critical care, Admission prediction.

## Abstract

**Background:**

Early identification of patients at risk for admission to the medical intensive care unit (MICU) at the time of medical admission is crucial for optimizing resource utilization and improving patient outcomes. No standardized, unified scoring system exists to predict MICU requirements for early medical admissions (EMA). This study aimed to develop and validate a predictive scoring system, the Medical Intensive Care Admission Prediction Score (MICAPS), to identify patients at high risk of transfer to the MICU based on demographic data, triage hemodynamics, and limited presentation-day laboratory data.

**Methods:**

This retrospective cross-sectional study included 11,847 adult patients admitted to medical floors via the emergency department (ED) at Hamad General Hospital, Qatar, between January 2019 and December 2019. Cerner^®^ was used to extract relevant data. Multivariate logistic regression identified significant predictors of MICU admission, and regression coefficients were used to develop the MICAPS model. ROC curve analysis and bootstrapping methods were employed to validate the model’s performance and accuracy.

**Results:**

Of 11,847 patients admitted to medical services, 909 (7.7%) were transferred to MICU. Significant predictors included male gender (OR: 1.41, 95% CI: 1.17-1.70), age ≤ 60 years (OR: 2.15, 95% CI: 1.72–2.68), abnormal respiratory rate (OR: 2.35, 95% CI: 1.48–3.72), oxygen saturation < 88% (OR: 1.95, 95% CI: 1.30–2.92), Glasgow Coma Scale < 9 (OR: 6.54, 95% CI: 4.91–8.71), RRT activation (OR: 3.82, 95% CI: 3.19–4.56), and abnormal laboratory values such as WBC ≥ 10 (OR: 1.29, 95% CI: 1.08–1.54) and lactate > 1.7 mmol/L (OR: 1.96, 95% CI: 1.64–2.34). MICAPS demonstrated good predictive power, with an area under the ROC curve of 0.809 (95% CI: 0.79–0.82), a sensitivity of 67.4%, a specificity of 81.3%, and a positive likelihood ratio of 3.60 at a score of ≥ 40.

**Conclusion:**

MICAPS is a simple-to-apply scoring system that enables the identification of patients early in their hospitalization who may require ICU care later during the hospital stay. It can support early clinical decision-making and optimize resource allocation in emergency departments, medical floors, and critical care settings. Further multicenter prospective validation is needed to assess its generalizability in the real world.

**Clinical trial number:**

Not applicable.

**Supplementary Information:**

The online version contains supplementary material available at 10.1186/s12873-025-01448-w.

## Introduction

Healthcare systems are experiencing a persistent rise in medical admissions around the globe [1]. This upward trend is multifactorial but thought to be primarily driven by aging populations, increasing multimorbidity, and enhanced access to care [2]. Interestingly, studies have shown that due to patients’ increasing complexities, there is a disproportionate increase in medical admissions compared to surgical or psychiatric admissions [3]. In recent years, multimorbidity among medical patients has increased up to 50%, and polypharmacy has surged by more than 80%, adding to the intricacy in medical care [3]. Consequently, frontline clinicians now face higher clinical complexity than ever before. A subgroup of patients admitted to the medical floors may require escalation of clinical care to ICUs in the first 24 to 48 h of admission [4].

ICUs are also facing significant pressure due to rising patient volumes amid limited resources. According to a 2024 CDC report, approximately 2.8 million emergency department (ED) visits result in admission to critical care units in the USA each year, accounting for about 15% of hospital admissions. ICU-related healthcare costs rose from $56.6 billion in 2000 to $108 billion in 2010, constituting 13.2% of hospital expenditures and 4.1% of national health expenditures [5, 6]. Although ICU bed occupancy and increasing complex medical presentations in the emergency departments (ED) are factors associated with the buildup of this pressure, the prolonged ED boarding times and delayed decision making for potential ICU candidates carry the worst patient outcomes [7]. Additionally, unplanned transfers to the ICU from medical wards are associated with worse patient-related outcomes, including ICU length of stay (LOS) and mortality [8]. Incorporating clinical decision-support tools to identify patients at risk of early deterioration during their admission can help overcome some of the challenges in patient care in fast-paced medical settings.

Traditional early warning scores (EWS), such as the Modified Early Warning Score (MEWS), National Early Warning Score (NEWS), and Rapid Emergency Medicine Score (REMS), were designed to identify patients at risk of deterioration in the ED. While they provide a fair assessment using minimal information, such as vital signs, current evidence highlights limitations in their persistent specificity in predicting ICU admission, especially for already admitted patients [9]. Additionally, these scoring systems are validated for early detection of clinical deterioration; however, they are specifically validated to pre-empt ICU needs, especially before or during any significant deterioration [9]. Furthermore, in recent decades, many scoring systems have been developed and validated to assess the severity of various medical conditions such as CURB-65 for pneumonia, Pulmonary Embolism Severity Index (PESI) for PE, bedside index of severity in acute pancreatitis (BISAP) score for acute pancreatitis and SOFA and SIRS for sepsis among others [10–14] While these scoring models remain essential for diagnosing, prognosticating, and predicting mortality in specific medical conditions, they are not designed or validated to predict ICU admission for early medical admissions (EMA). Their lack of generalizability across undifferentiated medical admissions leaves a critical gap in universal, early risk assessment for ICU needs. Many of these models integrate several laboratory parameters, such as white blood cell count, serum glucose, serum lactate, serum bilirubin, serum electrolytes, creatinine, and acid-base disturbances. Additional biomarkers, including C-reactive protein, B-type natriuretic peptide, and coagulation-related markers, have emerged as valuable tools for risk stratification and outcome prediction in at-risk patients [15]. However, for multiple reasons, incorporating multiple biomarkers in scoring systems to predict ICU admissions in EMAs might not be practical. For instance, this can delay decision-making in already fast-paced settings, significantly increase healthcare costs, and limit the generalizability of models in resource-limited healthcare settings. Therefore, simplified models based on limited, readily available triage data are needed to accurately predict ICU needs in at-risk EMAs.

Despite the critical need, literature lacks validated tools to predict ICU need within the first 24 to 48 h of medical admission using cost-effective, readily accessible data such as triage parameters, demographics, and a limited set of basic laboratory investigations. This study aimed to develop and internally validate the Medical Intensive Care Admission Prediction Score (MICAPS), a scoring system that uses demographic data, triage hemodynamics, and minimal day-1 basic laboratory parameters to identify early medical admissions at high risk of subsequent MICU transfer, thereby optimizing healthcare.

## Materials and methods

### Study design and setting

A single-center, retrospective, cross-sectional study was conducted, with data extracted from electronic medical records (EMRs) using the Health Information Management (HIM) patient identifiers. The extracted data encompassed patient demographics, comorbid conditions, hemodynamics at presentation, information regarding a recent hospital discharge, intensive care admission rates, and in-hospital mortality during the relevant admission.

### Study population

We collected and analyzed pre-specified data from all consecutive adult patients admitted to the medicine service from the emergency department (ED) between January 1, 2019, and December 31, 2019, who fulfilled the study inclusion criteria. We chose the 2019 dataset to intentionally avoid potential confounding introduced by the COVID-19 pandemic, which dramatically altered ED presentations, Medical admissions, ICU triage patterns, patient demographics, and thresholds for clinical care escalations worldwide. Using a pre-pandemic dataset allows the model to be developed on a more “baseline” representation of medical admissions. Patients were divided into two groups: those subsequently admitted to the medical intensive care unit (MICU) and those who remained on medical floors. For data collection in this study, ‘Early Medical Admissions (EMA)’ was defined as patients admitted under general internal medicine services within the first 24 h of hospital presentation.

### Inclusion criteria

Patients aged 14 years or older who were admitted to the medicine service via the emergency department during the study period were included. Patients aged ≥ 14 years were included as 14 years is the nationally accepted threshold for paediatric-to-adult care in Qatar (the country of study) and other GCC countries with similar patient populations [16].

### Exclusion criteria

Patients who died or were discharged before being admitted to the medical floor were excluded.

### Primary outcome

The study’s primary outcome was to develop a scoring system to predict MICU admission using demographic and clinical information, hemodynamic data at E.D. presentation, and laboratory parameters on day 1 of presentation.

### Statistical analysis

Descriptive statistics were used to summarize participants’ characteristics and to assess data distribution. Normally distributed data were presented as the mean ± standard deviation (SD), whereas the median and interquartile range (IQR) were used for skewed or non-normal distributions. Categorical data were summarized using frequencies and respective percentages. Associations between two or more qualitative variables were assessed using the Chi-square (χ2) test, Fisher’s Exact or Yates’ corrected Chi-square tests as appropriate. We analyzed quantitative data between the two independent groups (MICU admission vs. non-MICU admission) using an unpaired t-test. A non-parametric Mann-Whitney U test was applied when the data distribution was skewed. The final analysis excluded patients with missing critical predictor data (such as vital signs, laboratory results). Missing data were < 5% for all included variables, so complete case analysis was deemed appropriate without imputation.

Univariate and multivariate logistic regression methods were used to assess the predictive value of each predictor or risk factor (such as age, gender, R.R., SPO2, GCS, systolic blood pressure, number of comorbidities, RRT, and laboratory parameters) for the binary outcome variable of MICU admission (yes/no). For multivariate logistic regression models, predictors were considered statistically significant (*P* < 0.10) in univariate analysis or deemed clinically meaningful. The results of logistic regression analyses were reported as odds ratios (ORs) along with associated 95% confidence intervals (CIs). The number of risk factors identified in multivariable logistic regression analyses was used to derive weighted risk scores, yielding a clinically applicable decision-making rule for predicting MICU admission. The regression coefficients were divided by the smallest coefficient and rounded to the nearest integer to derive a simple-to-compute risk score.

The primary data analysis in our research study aimed to evaluate the predictive accuracy of various potential predictors and covariates (demographic and clinical information, hemodynamics at the E.D. presentation, and laboratory parameters on day 1 of presentation) for MICU admission using a developed weighted risk score classification. The sensitivity, specificity, positive and negative likelihood ratios, and accuracy indices for these parameters were calculated using admission to the MICU as the reference standard. A receiver operating characteristic (ROC) curve was calculated using significant predictors (as determined by multivariate logistic regression) to derive optimal cut-off values and assess the regression model’s discrimination and predictive accuracy. Because sensitivity and specificity were considered equally important, the best cut-off points were determined using Youden’s index, which maximizes sensitivity and specificity.

The bootstrapping (re-sampling) method, introduced by Efron (1979), was used to calculate bias-corrected percentile intervals (BCa) using 100 re-samples, thereby making a traditional multivariate regression model a more realistic representation of the population. All statistical analyses were performed using SPSS version 29.0 (Armonk, NY: IBM Corp) and Epi-Info (Centers for Disease Control and Prevention, Atlanta, GA) software. A two-sided P-value < 0.05 was considered statistically significant. The statistical analysis for this study was performed by the same statisticians who performed the statistical analysis for other research studies in which a scoring system for foreign body aspiration in children and admission to the medical ward (MAPS) was evaluated and formulated [17, 18]. The same statistical methods were used to develop a potential scoring system to predict MICU admission in this study.

### Ethics declaration

The Medical Research Centre (MRC) approved the study at Hamad Medical Corporation, Qatar, with approval ID MRC-01-20-1094.

### Participant consent

The IRB waived informed consent because this study was a retrospective review of medical records.

## Results

### Baseline demographics

Of the 11,847 patients admitted to medical floors from the emergency department (E.D.), 909 (7.7%) required ICU admission. The mean age of the study cohort was 49.74 ± 19.17 years, with no significant difference between those admitted to the ICU and those who were not (49.70 ± 19.25 vs. 50.13 ± 18.20 years, *p* = 0.503). Males constituted 56.8% (6733) of the total cohort, with a significantly higher preponderance for ICU admission in males compared to females (618/6733; 9.2% vs. 291/5114; 5.7%, *p* < 0.001) (Table 1).

**Table 1:** Baseline characteristics of all E.D. Admitted cases and comparison of ICU Admitted vs. non-ICU Admitted patients (*N* = 11847)

Baseline Characteristics	Study Cohort	ICU Admission	Non-ICU Admission	*p*-value
**Total Number**	11,847	909 (7.7%)	10,938 (92.3%)	
**Age (years) (Mean ± SD)**	49.74 ± 19.17	49.70 ± 19.25	50.13 ± 18.20	0.503
**Age Group** ≤ 60 years > 60 years	8247 (69.6%) 3600 (30.4%)	643 (70.7%) 266 (29.3%)	7604 (69.5%) 3334 (30.5%)	0.440
**Gender** Male Female	6733 (56.8%) 5114 (43.2%)	618 (68%) 291 (32%)	6115 (55.9%) 4823 (44.1%)	< 0.001
**Vitals**				
**Temperature (°C)**	36.97 ± 0.70	36.94 ± 0.77	36.97 ± 0.69	0.374
**Respiratory Rate (breaths/min)** 12–24 10–11 or 24–34 < 9 or > 35	19.71 ± 5.48	21.24 ± 8.46	19.56 ± 5.42	< 0.001
	10,939 (92.3%) 733 (6.2%) 147 (1.2%)	713 (78.4%) 131 (14.4%) 60 (6.6%)	10,226 (93.5%) 602 (5.5%) 87 (0.8%)	
**Pulse Rate (beats/min)** 50–100 101–110 111–130 or 41–50 > 130 or < 40	91.34 ± 24.89	95.41 ± 23.47	90.76 ± 19.27	< 0.001
	8375 (70.7%) 1440 (12.2%) 1559 (13.2%) 440 (3.7%)	552 (60.7%) 105 (11.6%) 154 (17%) 87 (9.6%)	7823 (71.5%) 1335 (12.2%) 1405 (12.8%) 353 (3.2%)	
**SpO₂ (%)** > 94 88–94 < 88	97.87 ± 3.43	96.57 ± 6.17	97.97 ± 3.08	< 0.001
	10,883 (91.9%) 722 (6.1%) 238 (2%)	762 (83.8%) 81 (8.9%) 66 (7.3%)	10,121 (92.5%) 641 (5.9%) 172 (1.6%)	
**GCS** 15 13–14 10–12 < 9	14.50 ± 1.86	12.43 ± 4.34	14.67 ± 1.34	< 0.001
	10,562 (89.2%) 463 (3.9%) 415 (3.5%) 398 (3.4%)	584 (64.2%) 74 (8.1%) 60 (6.6%) 191 (21%)	9978 (91.3%) 389 (3.6%) 355 (3.3%) 207 (1.9%)	
**Systolic BP (mmHg)** 100–160 160–180 or 81–100 180–220 or 71–80 > 220 or < 70	132.60 ± 28.58	133.34 ± 41.74	132.54 ± 27.21	0.570
	10,187 (86%) 944 (8%) 593 (5%) 120 (1%)	711 (78.2%) 89 (9.8%) 77 (8.5%) 31 (3.4%)	9476 (86.7%) 855 (7.8%) 516 (4.7%) 89 (0.8%)	
**Comorbidities**				
**IHD**	1303 (11%)	126 (13.9%)	1177 (10.8%)	0.004
**CVA/TIA**	2028 (17.1%)	282 (31%)	1746 (16%)	< 0.001
**Hypertension**	6172 (52.1%)	569 (62.6%)	5603 (51.2%)	< 0.001
**Diabetes**	5579 (47.1%)	425 (46.7%)	5154 (47.2%)	0.800
**CKD**	2623 (22.1%)	240 (26.4%)	2383 (21.8%)	0.001
**COPD**	851 (7.2%)	72 (7.9%)	779 (7.1%)	0.370
**Malignancy**	1228 (10.4%)	123 (13.5%)	1105 (10.1%)	0.001
**Chronic Lung Disease (Unspecified)**	2030 (17.1%)	150 (16.5%)	1880 (17.2%)	0.590
**Discharged within the last 30 days**	2499 (20.7%)	225 (24.8%)	2274 (20.8%)	0.005
**Number of Comorbidities (Mean ± SD)** **0** **1–2** **3–4** **> 5**	2.17 ± 1.86	2.38 ± 1.83	2.05 ± 1.85	< 0.001
	3316 (28%) 3956 (33.4%) 3188 (27%) 1387 (11.7%)	160 (17.6%) 360 (39.6%) 273 (30%) 116 (12.8%)	3156 (28.9%) 3596 (32.9%) 2915 (26.7%) 1271 (11.6%)	

RR: Respiratory Rate; PR: Pulse Rate; SPO2: Oxygen saturation; GCS: Glasgow Coma Scale; B.P.: Blood pressure; CKD: Chronic Kidney Disease; COPD: Chronic Obstructive Pulmonary Disease; SD: Standard Deviation. Percentages were calculated against the total number of each predictor subcategory, and all percentages (%) were computed using non-missing data values

### Baseline hemodynamic parameters and comorbidity burden

Compared to non-ICU patients, those requiring ICU care exhibited significantly worse hemodynamic profiles: higher respiratory rate (*p* < 0.001), lower mean oxygen saturation (*p* < 0.001), and lower mean Glasgow Coma Scale (GCS) (*p* < 0.001). Systolic blood pressure was comparable (*p* = 0.570). The prevalence of comorbidities, including IHD, CVA/TIA, hypertension, CKD, and malignancy, was significantly higher in ICU patients (all *p* < 0.05**)** (Table 1). The descriptive values of various laboratory parameters between ICU-admitted and non-ICU-admitted groups have been demonstrated in Supplementary Table 1.

### Risk factors for ICU admission

Univariate logistic regression analysis identified several key potential predictors and risk factors for ICU transfer among EMAs. These included male gender and extremes of respiratory rate (RR), pulse rate (PR), and systolic blood pressure (SBP), with a temporal relationship to ICU transfer. Similarly, lower oxygen saturation (SPO^2^) and Glasgow Coma Scale (GCS) were associated with significantly increased odds of ICU transfer. Most comorbidities were associated with ICU transfer. Among comorbidities, 1–2 conditions carried the highest odds of ICU admission, followed by 3–4 and ≥ 5 (Table 2.1). Notably, age was not a significant predictor in univariate analysis. Rapid response team (RRT) activation and a discharge within the last 30 days were also associated with ICU transfer.

**Table 2.1:** Univariate logistic regression analysis of predictors associated with ICU admission

Predictors	OR (95% CI)	*p*-value
Male gender	1.68 (1.45–1.94)	< 0.001
**Age** ≤ 60 years	1.06 (0.91–1.23)	0.443
**Respiratory Rate** 10–11 or 24–34 < 9 or > 35	3.12 (2.55–3.83) 9.89 (7.06–13.86)	< 0.001 < 0.001
**Pulse Rate** 101–110 111–130 or 41–50 > 130 or < 40	1.12 (0.89–1.38) 1.55 (1.29–1.87) 3.49 (2.72–4.49)	0.326 < 0.001 < 0.001
**SpO₂** 88–94% < 88%	1.68 (1.32–2.14) 5.10 (3.80–6.83)	< 0.001 < 0.001
**GCS** 13–14 10–12 < 9	3.25 (2.50–4.23) 2.89 (2.17–3.84) 15.77 (12.73–19.52)	< 0.001 < 0.001 < 0.001
**Systolic BP** 161–180 or 81–100 181–220 or 71–80 > 220 or < 70	1.39 (1.10–1.75) 1.99 (1.55–2.56) 4.64 (3.06–7.04)	0.006 < 0.001 < 0.001
**Comorbidities**		
**IHD**	1.33 (1.10–1.63)	0.004
**CVA/TIA**	2.37 (2.04–2.75)	< 0.001
**Hypertension**	1.59 (1.39–1.83)	< 0.001
**Diabetes**	0.98 (0.86–1.13)	0.832
**CKD**	1.29 (1.10–1.50)	0.001
**COPD**	1.12 (0.87–1.44)	0.370
**Malignancy**	1.39 (1.14–1.70)	0.001
**Chronic Lung Disease**	0.95 (0.79–1.14)	0.598
**Discharged within the last 30 days**	1.25 (1.07–1.47)	0.005
**Number of Comorbidities** 1–2 3–4 *≥* 5	1.98 (1.63–2.39) 1.85 (1.51–2.26) 1.80 (1.40–2.30)	< 0.001 < 0.001 < 0.001
**RRT Activation**	5.06 (4.40–5.83)	< 0.001
**Laboratory Parameters**		
**WBC ≥ 10 × 10³/µL**	2.30 (2.01–2.64)	< 0.001
**Creatinine > 80 µmol/L**	1.94 (1.68–2.23)	< 0.001
**Lactate > 1.7 mmol/L**	2.80 (2.40–3.26)	< 0.001
**CRP > 5 mg/L**	1.23 (1.03–1.46)	0.022

CI: Confidence interval; Percentages were calculated against the total number of each predictor subcategories and all percentages (%) were computed using non-missing data values

Univariate logistic regression was performed to assess the association between patient characteristics and ICU admission. Odds ratios (OR) and 95% confidence intervals (CI) are reported for each category relative to its designated reference group. For each variable, the following categories were used as the reference: Female gender, Age > 60 years, Respiratory rate 12–24 breaths/min, Pulse rate 50–100 beats/min, SpO₂ >94%, GCS 15, and Systolic BP 101–160 mmHg. P-values < 0.05 were considered statistically significant

Readily available laboratory parameters from the first 24 h of admission were significantly associated with ICU transfer in EMAs. Elevated white blood cell count (> 10 × 10³/µL), serum creatinine (> 80 µmol/L), lactate (> 1.7 mmol/L), and C-reactive protein (> 5 mg/L) were associated with significantly higher odds of ICU admission (Table 2.1). Table 2.2 presents the odds of ICU transfer based on the laboratory parameters analyzed in their original continuous form. Differences in laboratory parameters between ICU-transferred and non-transferred groups are shown in Supplementary Table 1.

**Table 2.2:** Lab parameters associated with ICU admission in the study cohort

Predictors (with Reference Range)	Reference Range	Unadjusted Odds Ratio (OR) (95% CI)	*P* value
WBC (x10³/µL)	4–10	1.03 (1.02–1.04)	< 0.001
Hemoglobin (gm/dL)	12–15	0.98 (0.96–1.01)	0.28
Platelets (x10³/µL)	150–410	0.99 (0.998–0.999)	< 0.001
Urea (mmol/L)	2.5–7.8	1.03 (1.02–1.03)	< 0.001
Creatinine (µmol/L)	44–80	1.001 (1-1.001)	< 0.001
Sodium (mmol/L)	133–146	0.99 (0.98-1)	0.26
Potassium (mmol/L)	3.5–5.3	1.05 (0.95–1.16)	0.36
Bicarbonate (28 mmol/L)	22–29	0.89 (0.88–0.91)	< 0.001
POC Glucose ( mmol/L)	3.5–5.5	1.001 (0.99–1.01)	0.9
Lactate (mmol/L)	0.5–2.2	1.4 (1.35–1.45)	< 0.001
Bilirubin (µmol/L)	3.4–20.5	1.004 (1.001–1.006)	0.003
AST (U/L)	0–55	1.001 (1.001–1.001)	< 0.001
ALT (U/L)	5–34	1.001 (1-1.001)	< 0.001
CRP (mg/L)	0–5	1.001 (1.001–1.002)	< 0.001
Procalcitonin (ng/mL)	< 0.5	1.022 (1.014–1.03)	< 0.001

### Development of the ICU admission risk score

**Fig. 1:**
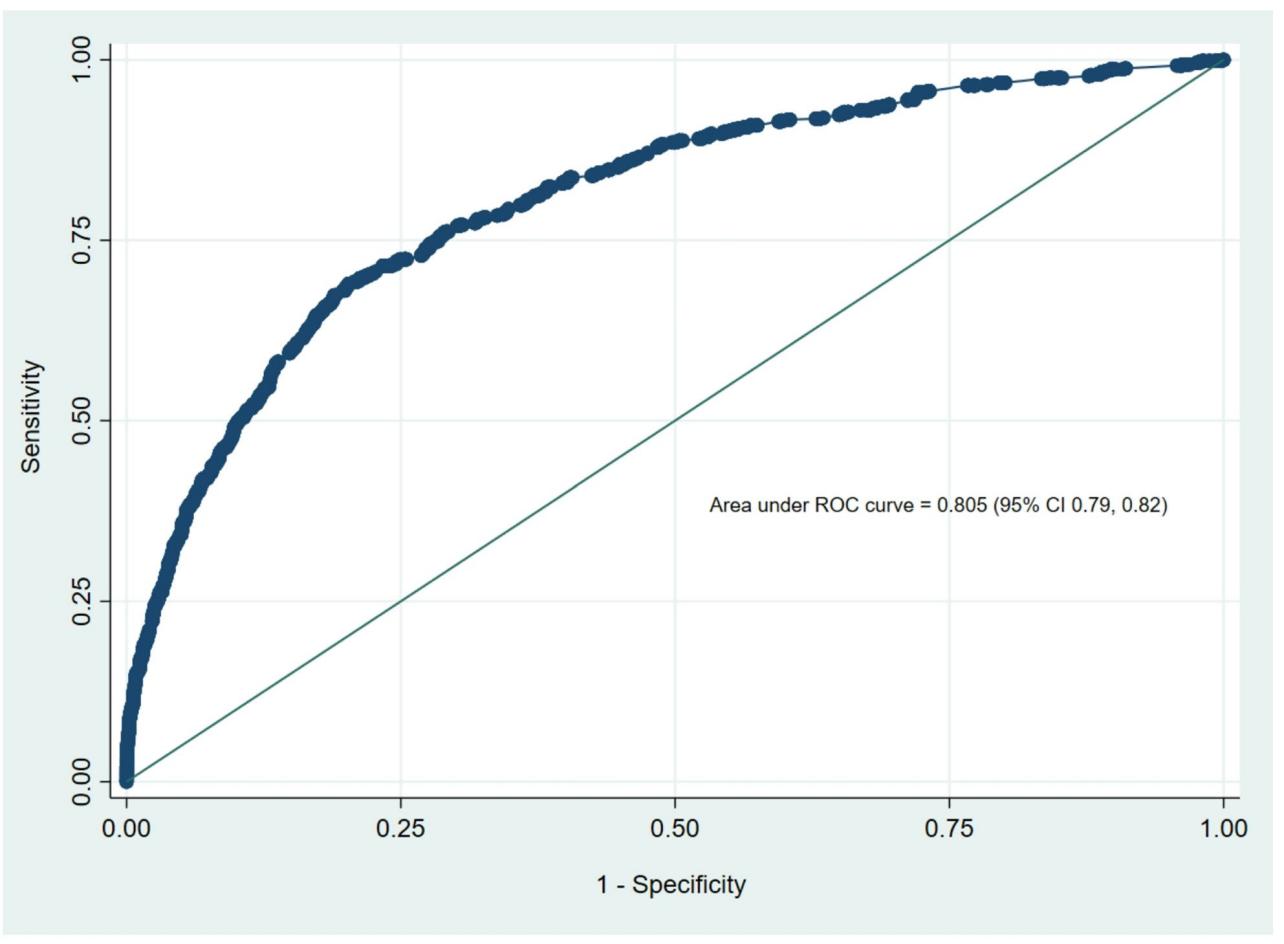
Predictive accuracy evaluation of the developed multivariate logistic regression model using ROC curve indices

**Fig. 2:**
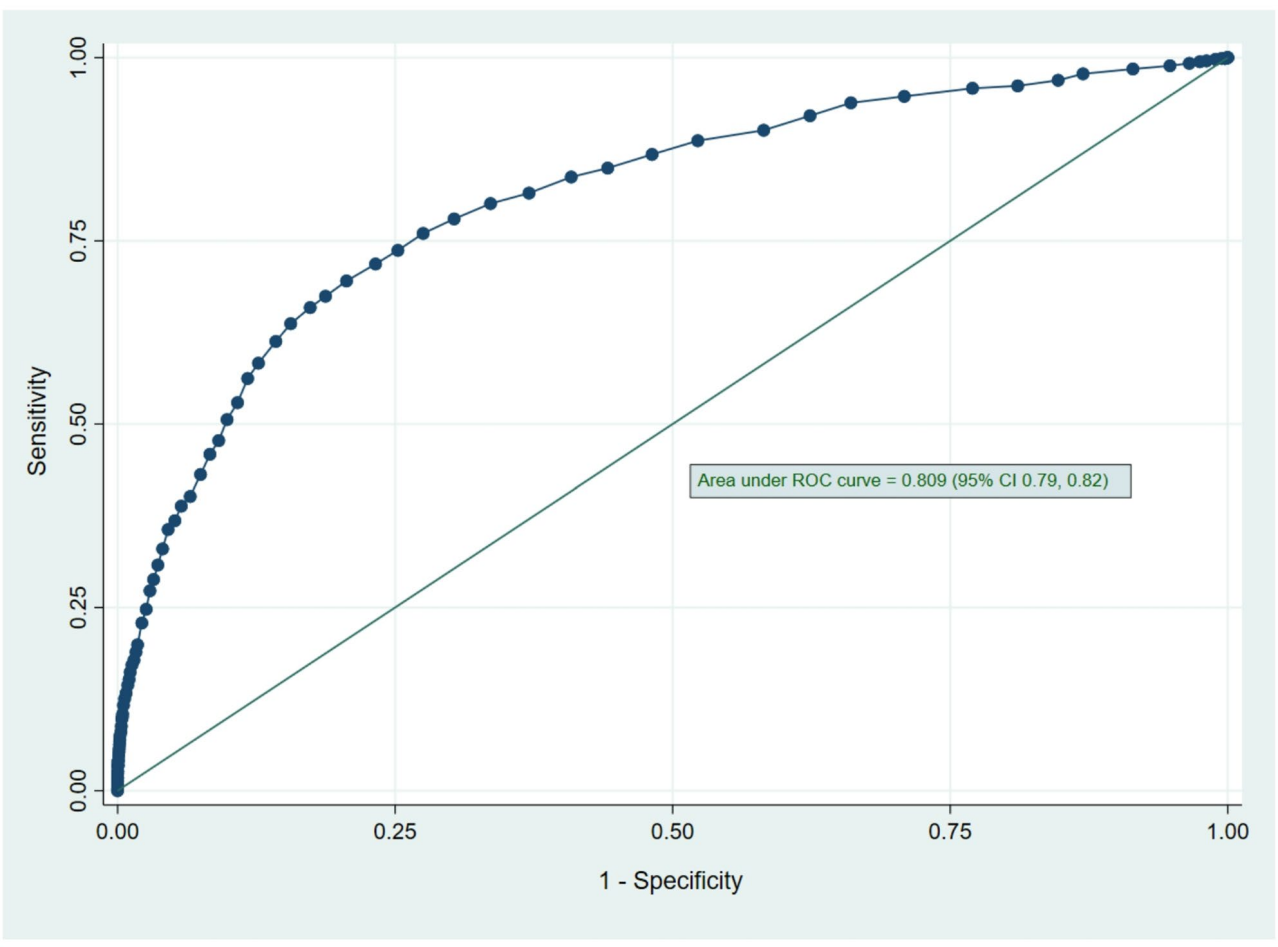
Receiver operating characteristic (ROC) curve of the ICU admission risk prediction score

A multivariate logistic regression model was developed to formulate a predictive scoring system for ICU transfer. The model included clinically and statistically significant predictors, including age, gender, hemodynamic status at ED presentation, number of comorbidities, RRT activation, and laboratory parameters. After multivariate regression, factors that retained significance in predicting ICU transfer included male gender and extremes of RR and SBP. Furthermore, lower SpO2 and GCS also remained significant predictors of ICU transfer. A higher number of comorbidities (except ≥ 5) also continued to be significant predictors of ICU transfer. Activation of the rapid response team (RRT) also remained a strong predictor of ICU transfer (OR 3.82, 95% CI 3.19–4.56; *p* < 0.001). Elevated white blood cell count, creatinine, and lactate remained significant predictors of ICU transfer (Table 3). Although age ≤ 60 years was not significantly associated with ICU transfer in univariate analysis, it emerged as an independent predictor in the multivariate model (adjusted odds ratio, 2.15; 95% confidence interval, 1.72–2.68; *p* < 0.001). The area under the receiver operating characteristic (ROC) curve for the developed multivariate logistic regression model was 0.805 (95% CI 0.79–0.82), indicating good predictive accuracy (Fig. 1). The Medical Intensive Care Transfer Prediction Score (MICAPS) was created using the above-mentioned potential predictors and factors. At a cut-off score of ≥ 35, the sensitivity was 78.0%, the specificity was 70.1%, with a positive likelihood ratio of 2.57, and an accuracy index value of 70.3%. Moreover, at a cut-off score of ≥ 40, the sensitivity was 67.4%, and the specificity was 81.3%, with a positive likelihood ratio of 3.60 and an accuracy index value of 80.2%. In addition, the various cut-off scores and their corresponding accuracy index values were evaluated and presented in Table 4. The area under the ROC curve for the MICAPS was 0.809 (95% CI 0.79–0.82) (Fig. 2). The MICAPS demonstrated robust performance across various cut-off points, as shown in Table 4. The statistical distribution of MICAPS, as shown in Supplementary Fig. 1, indicates that a substantial proportion of patients had MICAPS ranging from 20 to 40, with a small proportion having MICAPS exceeding 60 or less than 15. Furthermore, the Box plot (Supplementary Fig. 2) shows the distribution of MICAPS across gender and age groups, indicating that MICAPS were higher in males than in females in both age groups (age ≤ 60 years and age > 60 years).

**Table 3:** Multivariate logistic regression model for determining significant potential predictors and deriving weighted risk scores for ICU admission

Predictors	Adjusted Odds Ratio (95% CI)	*P*-value	Weight Risk score
Male gender	1.41 (1.17–1.70)	< 0.001	5
Age ≤ 60 years	2.15 (1.72–2.68)	< 0.001	10
R.R. (breaths pm) 10–11 or 24–34 *≤* 9 or *≥* 35	1.34 (1.02–1.76) 2.35 (1.48–3.72)	< 0.001 < 0.001	4 11
SpO2 (%) 88–94 < 88	0.93 (0.69–1.25) 1.95 (1.30–2.92)	0.623 0.001	1 9
GCS 13–14 10–12 < 9	2.23 (1.61–3.10) 1.55 (1.08–2.23) 6.54 (4.91–8.71)	< 0.001 0.018 < 0.001	11 6 25
Systolic B.P. (mmHg) 161–180 or 81–100 181–220 or 71–80 > 220 or < 70	1.31 (0.96–1.80) 1.70 (1.17–2.46) 4.10 (2.01–8.43)	0.093 0.005 < 0.001	4 7 19
Number of Comorbidities 1–2 3–4 *≥* 5	1.56 (1.23–2.03) 1.51 (1.14–2.01) 1.30 (0.91–1.85)	< 0.001 0.004 0.146	6 5 4
RRT activated	3.82 (3.19–4.56)	< 0.001	18
WBC (103/uL) ≥ 10	1.29 (1.08–1.54)	0.005	3
Creatinine (umol/L) > 80	1.48 (1.23–1.79)	< 0.001	5
Lactate (mmol/L) > 1.7	1.96 (1.64–2.34)	< 0.001	9

RR: Respiratory Rate; PR: Pulse Rate; SPO2: Oxygen saturation; GCS: Glasgow Coma Scale; BP: Blood pressure; SD: Standard Deviation; OR: odds ratio; CI: confidence interval

Adjusted ORs and 95% CI from multivariate logistic regression are presented for factors associated with ICU admission. All predictors were adjusted for other potential covariates found significant in the univariate logistic regression analysis. Weighted risk scores were computed by dividing the regression coefficients of the included predictors by the smallest regression coefficient and then rounding to the nearest integer. A total score was calculated for each patient by adding the scores corresponding to their characteristics. For categorical variables, the following reference groups were used: Female gender, Age ≤ 60 years, Respiratory rate 12–24 breaths/min, SpO₂ >94%, GCS 15, Systolic BP 101–160 mmHg, Number of Comorbidities = 0, No RRT activation, WBC < 10 × 10³/µL, Creatinine ≤ 80 µmol/L, and Lactate ≤ 1.7 mmol/L. P-values < 0.05 were considered statistically significant

**Table 4:** Predictive accuracy across different weighted risk score cut-off

ICU Admission Risk Score	Sensitivity	Specificity	Correctly Classified	LR^+^	LR^−^
≥ 30	86.8%	51.9%	54.5%	1.81	0.25
≥ 35	78.0%	70.1%	70.3%	2.57	0.32
≥ 40	67.4%	81.3%	80.2%	3.60	0.41
≥ 45	56.2%	88.3%	85.8%	4.80	0.50
≥ 50	43.1%	92.5%	88.7%	5.77	0.61

LR^+^: Likelihood ratio of a Positive Test, L.R^−^: Likelihood ratio of a Negative Test

To assess the predictability of ICU transfer using readily available clinical information at triage, an ROC curve was plotted, excluding laboratory parameters, which showed an area under the curve of 0.79 (95% CI, 0.78–0.81) (Supplementary Fig. 3).

### Validation of the ICU admission risk score

The developed regression model was validated using the bootstrapping (resampling) method, and bias and Bootstrap 95% C.I. were presented. One hundred resamples were used to make a traditional multivariate regression model a realistic model [19]. Based on the sample results, bootstrap confidence intervals were narrower than those of the developed traditional logistic regression model. The bootstrap-corrected model had less than 0.05% bias across all predictor parameters relative to the sample traditional model coefficients, as shown in Table 5.

**Table 5:** Bootstrap validation of final model predictors

Predictor	Bias	SE (Corrected)	Bootstrap 95% CI (Lower–Upper)
Male Gender	0.006	0.045	1.26–1.60
R.R. 10–11 or 25–34	0.009	0.059	1.82–2.61
R.R. <9 or > 35	0.019	0.084	3.33–7.27
SpO2 88–94%	0.013	0.065	1.24–1.98
SpO2 < 88%	0.027	0.094	3.06–5.84
GCS 13–14	0.012	0.062	1.80–3.10
GCS 10–12	0.018	0.070	1.89–3.51
GCS < 9	0.036	0.110	8.09–14.90
SBP 71–80 or 181–220	0.015	0.068	1.43–2.58
SBP > 220 or < 70	0.028	0.098	2.36–5.60
RRT Activation	0.014	0.056	3.30–4.60
WBC ≥ 10 × 10^3/uL	0.012	0.054	1.38–1.83
Lactate > 1.7 mmol/L	0.016	0.058	1.53–2.14
Creatinine > 80 µmol/L	0.008	0.051	1.10–1.51

Bootstrap validation (100 resamples) assessed the stability of the multivariate logistic regression model predicting ICU admission in the study cohort. Bias, corrected standard error (SE), and bootstrap-derived 95% confidence intervals (CI) are reported for each predictor. All predictors demonstrated minimal bias (close to 0), confirming stability across resampling iterations.​ Low SE and Narrow confidence intervals (CIs) reflect model reliability

## Discussion

This study analyzed data from a diverse cohort of more than 10,000 patients (in the first few hours of hospitalization) with medical diagnoses to develop and validate the Medical Intensive Care Admission Prediction Score (MICAPS). MICAPS requires readily accessible clinical and laboratory data, regardless of a healthcare setting’s resources, including patient demographics, triage hemodynamic status, comorbidity burden, and select biomarkers, to predict ICU need. Medical diagnosis was not incorporated to maintain MICAPS’ universality for all medical admissions. The model generating MICAPS demonstrated excellent discriminatory ability with an AUC of 0.81. The model was then validated by generating 100 bootstrap resamples. Minimal bias and low standard errors across resampling iterations confirmed the model’s stability and reliability. The results of this study emphasize the utility of a rapid, resource-conscious, and accurate risk-stratification model at the point of, or immediately after, admission to guide early clinical decision-making across diverse healthcare settings.

ICUs are among the most expensive units in hospitals, accounting for 20–30% of hospital care costs [20]. With the rapid evolution of medical technology and research, contributing to more specialized and precise treatments, shifts in disease epidemiology, and the growing complexity of patient cases, ICU resource costs continue to escalate [3]. Factors such as length of stay, utilization of ICU resources like mechanical ventilation, haemodialysis, and severity of illness are some of the many other drivers of ICU costs [21]. Consequently, managing flow across ICU occupancy is becoming increasingly complex and challenging for healthcare managers and policymakers [20, 22].

The timing of transfer to the ICU is a critical determinant of clinical outcomes in hospitalized patients. Delays can result in a higher degree of organ dysfunction, a prolonged ICU stay, and around 4-fold higher odds of mortality [23, 24]. Critical care provision is not limited to the ICU; advanced ICU care, such as non-invasive ventilation and inotropic support with monitoring, is available in non-ICU settings with adequate staff training [25]. However, it is imperative to identify high-risk groups promptly to provide timely critical care. Early ICU reviews with timely interventions can improve patient outcomes and optimize resource utilization, hence reducing costs to some extent [26]. Delayed ICU care, on the other hand, depends not only on the availability or capacity of an ICU setting but also on the experience and clinical judgment of treating physicians in recognizing a deteriorating patient and seeking help on time before the patient crashes [27]. A previous study reported that assessment by junior, less experienced staff was one of the reasons for delayed transfers to the ICU [28]. Utilizing an easily applicable yet comprehensive scoring system, such as MICAPS, can greatly assist physicians in enhancing patient monitoring, facilitating timely ICU reviews, and ensuring prompt interventions, ultimately improving daily practice outcomes.

Over the last two decades, alerting systems have been developed in healthcare facilities to detect deteriorating patients. Late recognition of patient deterioration is associated with poor outcomes, including higher mortality [29]. Most alerting systems use hemodynamic data (early warning scores - EWS), and rapid response teams respond to these alerts; however, the evidence is conflicting regarding whether these systems improve patient outcomes [30, 31]. Prediction models based on EWS, including NEWS, NEWS2, and MEWS, are physiological scoring systems developed and mandated in specific parts of the world to identify deteriorating hospitalized patients outside the critical care settings [32]. In the United Kingdom, the NEWS has been formally mandated across the National Health Service to trigger timely interventions when patients show signs of clinical deterioration [33]. However, these scoring systems have inherent limitations and may not be equally valid for use beyond identifying patients who are deteriorating. One comparative study that assessed six different EWS for their ability to predict ICU admission or in-hospital mortality within 24 h of emergency department presentation found that NEWS was the most accurate predictor (area under the receiver operating characteristic [AUROC] curve, 0.904 [95% CI, 0.805–0.913]) [34]. However, a systematic review of the methodologies of these EWS concluded that they exhibit inconsistent calibration and poor specificity, leading to high rates of false-positive alarms and alarm fatigue. It also highlighted heterogeneity in threshold settings across healthcare settings, causing delayed recognition of actual deterioration and inappropriate escalation of care [35].

While clinical deterioration often prompts consideration of ICU admission, the decision is seldom straightforward and requires careful clinical judgement. Factors such as age, comorbidity burden, disease severity and salvageability, extent of involvement in other organ systems, and overall functional status must all be weighed in to determine whether the patient will truly benefit from intensive care [36]. Among the various factors in clinical decision-making, patient age remains one of the most frequently used yet most controversial factors [37]. The world’s population is indeed aging; in 2010, there were 524 million people above the age of 65, and this number is expected to increase to 1.5 billion by 2050 [38]. Advancements and increased access to healthcare have enabled the aging population with multiple comorbidities to live longer, resulting in an economic burden on healthcare systems [38]. A recent review showed that half of the patients admitted to the ICU are now in fact older than 65 years [39].

In this study, age ≤ 60 years was not significantly associated with ICU admission in univariate analysis, suggesting that, in isolation, younger age may not predict ICU transfer. However, in the multivariate model, after adjusting for factors such as GCS, SpO₂, respiratory rate, and comorbidities, age ≤ 60 emerged as a strong independent predictor of ICU admission compared with older patients. This highlights the impact of confounding in the univariate analysis, where the effect of age was likely masked by other overlapping risk variables that are more prevalent in older adults. Our cohort mainly comprised individuals *≤* 60 years of age (69.6%). Likewise, among those transferred to the ICU, 70.7% were *≤* 60 years of age. The most plausible explanation is Qatar’s population demographics. In 2020, the median age of Qatar’s population was 31.3 years. 75% were male, and 88% were expatriates, mainly from Southeast Asia [40]. Another explanation for younger patients having higher odds of ICU admission in this study (after adjusting for other severity indicators) is that clinicians may have perceived these patients as more salvageable, given their youth, fewer frailty-related comorbidities, and a higher likelihood of returning to baseline functionality. This paradigm became especially apparent during the COVID-19 pandemic, when the global ICU bed shortages compelled physicians to prioritize age as a factor to assess salvageability when deciding ICU resource allocations [41].

The prevalence of multimorbidity is increasing globally [42]. For instance, in the UK, one in three adults admitted to hospitals has five or more comorbid conditions [43]. Multimorbidity is associated with poorer functional status, reduced quality of life, and poorer health outcomes [42]. A study found that hospital mortality rates were 33% for patients with one comorbidity and 39% for those with two or more comorbidities, significantly higher than the rates for those without any comorbidities (25%) (*P* < 0.01) [44]. The presence of comorbidities and the patient’s functional status are key factors considered in the decision-making process for ICU admission [45]. A study showed that scoring systems combining comorbidities and acute physiologic parameters provide more accurate prognostic estimates than those based on either acute physiologic measures or long-term medical history [46]. Interestingly, in the current study, patients with 1–2 comorbidities had higher odds of ICU admission than those with ≥ 3 comorbidities. This likely reflects the complex intersection of clinical judgment and expected benefit. Patients with moderate comorbidity burden may be deemed suitable for escalation and deemed more salvageable. In contrast, those with multiple chronic illnesses often have treatment limitations or are considered poor ICU candidates due to frailty, end-stage disease, or limited reversibility [47].

The integration of laboratory tests with vital signs to formulate illness severity scores, such as APACHE IV and SAPS3, and predict outcomes is a well-established practice in clinical medicine. This provides healthcare professionals with a comprehensive understanding of the patient’s health, enabling them to make informed decisions in critical situations [48]. The use of specific laboratory tests as surrogate markers of certain medical conditions, such as troponins for acute coronary syndrome, is also well established [49]. However, the application of routine investigations at the time of ED presentation or after hospitalisation to predict deterioration or the need for ICU admission is less thoroughly studied. A study demonstrated that a combination of laboratory tests at presentation can provide predictive value regarding ICU admission or mortality within the same or the following day [50]. However, to ensure timely and broad applicability, especially in resource-limited healthcare setups, it is crucial to devise a clinical decision support system that uses minimal laboratory data to guide disposition decisions in fast-paced environments, as many patients continue to remain in the ED for many hours post-admission orders, contributing to ED crowding [51]. The MICAPS model incorporated the four most clinically relevant and readily available laboratory investigations (WBC, lactate, creatinine, and CRP) to predict ICU requirements immediately after a medical admission, before any clinical deterioration. By focusing on these readily obtainable biomarkers with established relevance in patients with deteriorating conditions, we enhanced the score’s generalizability while maintaining robust predictive performance.

The current study has many notable strengths, including the use of a large dataset to devise a novel and robust scoring system. Unlike prior alarm systems, which primarily rely on vital signs and often miss significant deteriorations, the MICAPS integrates the patient’s level of consciousness, past medical history, and minimal routine blood tests to create a framework for scoring. This framework is designed to identify, within hours of hospitalization, early medical admissions who may require ICU care during that hospitalization, even before any clinical deterioration occurs. MICAPS is a unified scoring system, regardless of the patient’s medical diagnosis at admission, that streamlines decision-making by providing a single, comprehensive risk estimate. This is particularly useful in busy medical floors and ED settings for making timely decisions. Another strength of this study is the ability to validate the model using bootstrapping, confirming model stability and predictive performance. Bootstrapping provided internal validation to the scoring system, improving confidence in its use for clinical decision-making. Additionally, the use of readily available triage data and patient demographics, along with minimal laboratory data, which is widely available and affordable in most parts of the world, makes the scoring model highly generalizable.

Nevertheless, the study has limitations, mainly inherent in its design. It is a single-centred retrospective study, suggesting inherent biases that could not be controlled. Although multivariate logistic regression can adjust for many variables, there may be hidden confounders that can only be controlled in a prospective design. The dataset captured patients admitted to the ICU, but did not capture those who were consulted for ICU admission but refused admission, which could have introduced selection bias. Admitting a patient to the ICU depends heavily on the treating physician’s clinical judgment and experience. Hence, despite MICAPS’ validation via bootstrapping, a prospective validation would be necessary to assess its real-world applicability. Additionally, this study did not compare outcomes between patients transferred to the ICU after medical admission (our cohort) and those admitted directly to the ICU from the ED. The lack of data on the time duration between admission to the medicine and transfer to the ICU, as well as the experience level of the physicians who made those decisions, are other notable limitations of this study. Although it was not part of the scoring system, because the data are subjective and difficult to extract, the impact of patients’ symptoms at presentation to the ED on MICAPS was not studied. Including patients aged 14–18 years might limit generalizability. However, subgroup analysis showed that this population did not influence the results (5.7% admitted to ICU with no statistical significance). While MICAPS utilizes objective, digitally extractable data, we acknowledge that frailty and functional status (often best captured through a comprehensive history) are central to ICU care decision-making. Unfortunately, our EMR system does not reliably encode frailty or functional status in structured fields that can be captured electronically; therefore, these variables were not extracted and analyzed.

## Conclusion

MICAPS is a simple-to-apply scoring system that enables the identification of patients early in their hospitalization who may later require ICU care. Healthcare professionals can utilize MICAPS to evaluate patients promptly, determine the appropriate monitoring frequency, and provide advanced care, even in non-ICU settings, if needed, pending transfer to the ICU. Further multicenter prospective validation is needed to assess its generalizability in the real world. Integrating MICAPS in real time with various electronic medical records will further streamline clinical workflows by creating a clinical decision support system.

## Supplementary Information

Below is the link to the electronic supplementary material.

Supplementary Material 1

## Data Availability

The datasets used and analysed in the current study are available from the corresponding author on reasonable request.

## Abbreviations

MICU: Medical Intensive Care Unit
EMA: Early Medical Admission
MICAPS: Medical Intensive Care Admission Prediction Score
ED: Emergency Department
OR: Odds Ratio
CI: Confidence Interval
ROC: Receiver Operating Characteristic
LOS: Length of Stay
EWS: Early Warning Score
MEWS: Modified Early Warning Score
NEWS: National Early Warning Score
REMS: Rapid Emergency Medicine Score
CURB-65: Confusion, Urea, Respiratory rate, Blood pressure, Age ≥ 65 score
PESI: Pulmonary Embolism Severity Index
PE: Pulmonary Embolism
BISAP: Bedside Index for Severity in Acute Pancreatitis
SOFA: Sepsis-related Organ Failure Assessment
SIRS: Systemic Inflammatory Response Syndrome
EMR: Electronic Medical Record
HIM: Health Information Department
S.D.: Standard Deviation
IQR: Interquartile Range
R.R.: Respiratory Rate
SPO2: Oxygen Saturation
GCS: Glasgow Coma Scale
B.P.: Blood Pressure
RRT: Rapid Response Team
SPSS: Statistical Package for the Social Sciences
MAPS: Medical Admission Prediction Score
IRB: Institutional Review Board
IHD: Ischemic Heart Disease
CVA/TIA: Cerebrovascular Accident / Transient Ischemic Attack
CKD: Chronic Kidney Disease
DM: Diabetes Mellitus
COPD: Chronic Obstructive Pulmonary Disease
SBP: Systolic Blood Pressure
CRP: C-reactive Protein
AUC: Area Under the Curve
AUROC: Area Under the Receiver Operating Characteristic
APACHE IV: Acute Physiology and Chronic Health Evaluation IV
SAPS3: Simplified Acute Physiology Score 3

## References

[1] Jones CH, Dolsten M. Healthcare on the brink: navigating the challenges of an aging society in the united States. NPJ Aging. 2024;10(1):22. 38582901 10.1038/s41514-024-00148-2 PMC10998868

[2] The Lancet Healthy. Care for ageing populations globally. Lancet Healthy Longev. 2021;2(4):e180. 34697611 10.1016/S2666-7568(21)00064-7 PMC8529576

[3] Naik H, Murray TM, Khan M, Daly-Grafstein D, Liu G, Kassen BO, Onrot J, Sutherland JM, Staples JA. Population-Based trends in complexity of hospital inpatients. JAMA Intern Med. 2024;184(2):183–92. 38190179 10.1001/jamainternmed.2023.7410 PMC10775081

[4] Henriksen DP, Brabrand M, Lassen AT. Prognosis and risk factors for deterioration in patients admitted to a medical emergency department. PLoS ONE. 2014;9(4):e94649. 24718637 10.1371/journal.pone.0094649 PMC3981818

[5] Critical Care Statistics. [https://www.sccm.org/communications/critical-care-statistics]

[6] de Lange DW, Soares M, Pilcher D. ICU beds: less is more? No. Intensive Care Med. 2020;46(8):1597–9. 32458052 10.1007/s00134-020-06089-0 PMC7248458

[7] Mathews KS, Durst MS, Vargas-Torres C, Olson AD, Mazumdar M, Richardson LD. Effect of emergency department and ICU occupancy on admission decisions and outcomes for critically ill patients. Crit Care Med. 2018;46(5):720–7. 29384780 10.1097/CCM.0000000000002993 PMC5899025

[8] Khanna AK, Moucharite MA, Benefield PJ, Kaw R. Patient characteristics and clinical and economic outcomes associated with unplanned medical and surgical intensive care unit admissions: A retrospective analysis. Clinicoecon Outcomes Res. 2023;15:703–19. 37780944 10.2147/CEOR.S424759 PMC10541084

[9] Spencer W, Smith J, Date P, de Tonnerre E, Taylor DM. Determination of the best early warning scores to predict clinical outcomes of patients in the emergency department. Emerg Med J. 2019;36(12):716–21. 31366627 10.1136/emermed-2019-208622

[10] Lim WS, van der Eerden MM, Laing R, Boersma WG, Karalus N, Town GI, Lewis SA, Macfarlane JT. Defining community acquired pneumonia severity on presentation to hospital: an international derivation and validation study. Thorax. 2003;58(5):377–82. 12728155 10.1136/thorax.58.5.377 PMC1746657

[11] Aujesky D, Obrosky DS, Stone RA, Auble TE, Perrier A, Cornuz J, Roy PM, Fine MJ. Derivation and validation of a prognostic model for pulmonary embolism. Am J Respir Crit Care Med. 2005;172(8):1041–6. 16020800 10.1164/rccm.200506-862OC PMC2718410

[12] Wu BU, Johannes RS, Sun X, Tabak Y, Conwell DL, Banks PA. The early prediction of mortality in acute pancreatitis: a large population-based study. Gut. 2008;57(12):1698–703. 18519429 10.1136/gut.2008.152702

[13] Vincent JL, Moreno R, Takala J, Willatts S, De Mendonça A, Bruining H, Reinhart CK, Suter PM, Thijs LG. The SOFA (Sepsis-related organ failure Assessment) score to describe organ dysfunction/failure. On behalf of the working group on Sepsis-Related problems of the European society of intensive care medicine. Intensive Care Med. 1996;22(7):707–10. 8844239 10.1007/BF01709751

[14] Kaukonen KM, Bailey M, Pilcher D, Cooper DJ, Bellomo R. Systemic inflammatory response syndrome criteria in defining severe sepsis. N Engl J Med. 2015;372(17):1629–38. 25776936 10.1056/NEJMoa1415236

[15] Howell MD, Donnino MW, Talmor D, Clardy P, Ngo L, Shapiro NI. Performance of severity of illness scoring systems in emergency department patients with infection. Acad Emerg Med. 2007;14(8):709–14. 17576773 10.1197/j.aem.2007.02.036

[16] Alotaibi Q, Dighe M. Assessing the need for pediatric palliative care in the six Arab Gulf Cooperation Council countries. Palliat Med Rep. 2023;4(1):36–40. 36910455 10.1089/pmr.2022.0037 PMC9994439

[17] Janahi IA, Khan S, Chandra P, Al-Marri N, Saadoon A, Al-Naimi L, Al-Thani M, Greer W. A new clinical algorithm scoring for management of suspected foreign body aspiration in children. BMC Pulm Med. 2017;17(1):61. 28407759 10.1186/s12890-017-0406-6 PMC5390464

[18] Zahid M, Khan AA, Ata F, Yousaf Z, Naushad VA, Purayil NK, Chandra P, Singh R, Kartha AB, Elzouki AYA, et al. Medical admission prediction score (MAPS); a simple tool to predict medical admissions in the emergency department. PLoS ONE. 2023;18(11):e0293140. 37948401 10.1371/journal.pone.0293140 PMC10637671

[19] Efron B. Bootstrap Methods: Another Look at the Jackknife. In: Breakthroughs in Statistics: Methodology and Distribution. edn. Edited by Kotz S, Johnson NL. New York, NY: Springer New York; 1992: 569–593.

[20] Wodchis WP, Austin PC, Henry DA. A 3-year study of high-cost users of health care. CMAJ. 2016;188(3):182–8. 26755672 10.1503/cmaj.150064 PMC4754179

[21] Mastrogianni M, Galanis P, Kaitelidou D, Konstantinou E, Fildissis G, Katsoulas T. Factors affecting adult intensive care units costs by using the bottom-up and top-down costing methodology in OECD countries: A systematic review. Intensive Crit Care Nurs. 2021;66:103080. 34059412 10.1016/j.iccn.2021.103080

[22] Bruyneel A, Larcin L, Martins D, Van Den Bulcke J, Leclercq P, Pirson M. Cost comparisons and factors related to cost per stay in intensive care units in Belgium. BMC Health Serv Res. 2023;23(1):986. 37705056 10.1186/s12913-023-09926-2 PMC10500739

[23] Young MP, Gooder VJ, McBride K, James B, Fisher ES. Inpatient transfers to the intensive care unit: delays are associated with increased mortality and morbidity. J Gen Intern Med. 2003;18(2):77–83. 12542581 10.1046/j.1525-1497.2003.20441.x PMC1494814

[24] Shibata J, Osawa I, Fukuchi K, Goto T. The association between time from emergency department visit to ICU admission and mortality in patients with sepsis. Crit Care Explor. 2023;5(5):e0915. 37181540 10.1097/CCE.0000000000000915 PMC10171575

[25] Weingart SD, Sherwin RL, Emlet LL, Tawil I, Mayglothling J, Rittenberger JC. ED intensivists and ED intensive care units. Am J Emerg Med. 2013;31(3):617–20. 10.1016/j.ajem.2012.10.015 23380127

[26] Dünser MW, Noitz M, Tschoellitsch T, Bruckner M, Brunner M, Eichler B, Erblich R, Kalb S, Knöll M, Szasz J, et al. Emergency critical care: closing the gap between onset of critical illness and intensive care unit admission. Wiener Klinische Wochenschrift. 2024;136(23):651–61. 38755419 10.1007/s00508-024-02374-w PMC11632058

[27] Peltonen L-M, McCallum L, Siirala E, Haataja M, Lundgrén-Laine H, Salanterä S, Lin F. An integrative literature review of organisational factors associated with admission and discharge delays in critical care. Biomed Res Int. 2015;2015(1):868653. 26558286 10.1155/2015/868653 PMC4629003

[28] Louriz M, Abidi K, Akkaoui M, Madani N, Chater K, Belayachi J, Dendane T, Zeggwagh AA, Abouqal R. Determinants and outcomes associated with decisions to deny or to delay intensive care unit admission in Morocco. Intensive Care Med. 2012;38(5):830–7. 22398756 10.1007/s00134-012-2517-0

[29] Pimentel MAF, Redfern OC, Malycha J, Meredith P, Prytherch D, Briggs J, Young JD, Clifton DA, Tarassenko L, Watkinson PJ. Detecting deteriorating patients in the hospital: development and validation of a novel scoring system. Am J Respir Crit Care Med. 2021;204(1):44–52. 33525997 10.1164/rccm.202007-2700OC PMC8437126

[30] Emergency and acute medical care in. over 16s: service delivery and organisation [https://www.nice.org.uk/guidance/ng94] 33270407

[31] McNeill G, Bryden D. Do either early warning systems or emergency response teams improve hospital patient survival? A systematic review. Resuscitation. 2013;84(12):1652–67. 23962485 10.1016/j.resuscitation.2013.08.006

[32] Fu LH, Schwartz J, Moy A, Knaplund C, Kang MJ, Schnock KO, Garcia JP, Jia H, Dykes PC, Cato K, et al. Development and validation of early warning score system: A systematic literature review. J Biomed Inf. 2020;105:103410. 10.1016/j.jbi.2020.103410 PMC7295317 32278089

[33] Williams B. The National early warning score: from concept to NHS implementation. Clin Med (Lond). 2022;22(6):499–505. 36427887 10.7861/clinmed.2022-news-concept PMC9761416

[34] Covino M, Sandroni C, Della Polla D, De Matteis G, Piccioni A, De Vita A, Russo A, Salini S, Carbone L, Petrucci M et al. Predicting ICU admission and death in the emergency department: a comparison of six early warning scores. Resuscitation 2023, volume 190. 10.1016/j.resuscitation.2023.109876 37331563

[35] Gerry S, Bonnici T, Birks J, Kirtley S, Virdee PS, Watkinson PJ, Collins GS. Early warning scores for detecting deterioration in adult hospital patients: systematic review and critical appraisal of methodology. BMJ. 2020;369:m1501. 32434791 10.1136/bmj.m1501 PMC7238890

[36] Sprung CL, Artigas A, Kesecioglu J, Pezzi A, Wiis J, Pirracchio R, Baras M, Edbrooke DL, Pesenti A, Bakker J et al. The Eldicus prospective, observational study of triage decision making in European intensive care units. Part II: intensive care benefit for the elderly*. Crit Care Med 2012, 40(1). 10.1097/CCM.0b013e318232d6b0 22001580

[37] Tranvåg EJ, Norheim OF, Ottersen T. Clinical decision making in cancer care: a review of current and future roles of patient age. BMC Cancer. 2018;18(1):546. 29743048 10.1186/s12885-018-4456-9 PMC5944161

[38] Global Health. and Aging [https://www.nia.nih.gov/sites/default/files/2017-06/global_health_aging.pdf]

[39] Boumendil A, Somme D, Garrouste-Orgeas M, Guidet B. Should elderly patients be admitted to the intensive care unit? Intensive Care Med. 2007;33(7):1252. 17404703 10.1007/s00134-007-0621-3

[40] Qatar. Average age of the population from 1950 to 2100 [https://www.statista.com/statistics/379519/average-age-of-the-population-in-qatar/]

[41] Persad G, Joffe S. Allocating scarce life-saving resources: the proper role of age. J Med Ethics. 2021;47(12):836–8. 10.1136/medethics-2020-106792 33753473

[42] Holland E, Matthews K, Macdonald S, Ashworth M, Laidlaw L, Cheung KSY, Stannard S, Francis NA, Mair FS, Gooding C, et al. The impact of living with multiple long-term conditions (multimorbidity) on everyday life – a qualitative evidence synthesis. BMC Public Health. 2024;24(1):3446. 39696210 10.1186/s12889-024-20763-8 PMC11654051

[43] Briefing. Understanding the health care needs of people with multiple health conditions [https://reader.health.org.uk/understanding-health-care-needs-people-multiple-health-conditions]

[44] Simpson A, Puxty K, McLoone P, Quasim T, Sloan B, Morrison DS. Comorbidity and survival after admission to the intensive care unit: A population-based study of 41,230 patients. J Intensive Care Soc. 2021;22(2):143–51. 34025754 10.1177/1751143720914229 PMC8120566

[45] Bassford CR, Krucien N, Ryan M, Griffiths FE, Svantesson M, Fritz Z, Perkins GD, Quinton S, Slowther AM. U.K. Intensivists’ preferences for patient admission to ICU: evidence from a choice experiment. Crit Care Med. 2019;47(11):1522–30. 31385883 10.1097/CCM.0000000000003903 PMC6798748

[46] Nielsen AB, Thorsen-Meyer HC, Belling K, Nielsen AP, Thomas CE, Chmura PJ, Lademann M, Moseley PL, Heimann M, Dybdahl L, et al. Survival prediction in intensive-care units based on aggregation of long-term disease history and acute physiology: a retrospective study of the Danish National patient registry and electronic patient records. Lancet Digit Health. 2019;1(2):e78–89. 33323232 10.1016/S2589-7500(19)30024-X

[47] Zador Z, Landry A, Cusimano MD, Geifman N. Multimorbidity States associated with higher mortality rates in organ dysfunction and sepsis: a data-driven analysis in critical care. Crit Care. 2019;23(1):247. 31287020 10.1186/s13054-019-2486-6 PMC6613271

[48] Zimmerman JE, Kramer AA, McNair DS, Malila FM. Acute physiology and chronic health evaluation (APACHE) IV: hospital mortality assessment for today’s critically ill patients. Crit Care Med. 2006;34(5):1297–310. 16540951 10.1097/01.CCM.0000215112.84523.F0

[49] Giannitsis E, Müller-Bardorff M, Lehrke S, Wiegand U, Tölg R, Weidtmann B, Hartmann F, Richardt G, Katus HA. Admission troponin T level predicts clinical outcomes, TIMI flow, and myocardial tissue perfusion after primary percutaneous intervention for acute ST-segment elevation myocardial infarction. Circulation. 2001;104(6):630–5. 11489766 10.1161/hc3101.093863

[50] Loekito E, Bailey J, Bellomo R, Hart GK, Hegarty C, Davey P, Bain C, Pilcher D, Schneider H. Common laboratory tests predict imminent medical emergency team calls, intensive care unit admission or death in emergency department patients. Emerg Med Australas. 2013;25(2):132–9. 23560963 10.1111/1742-6723.12040

[51] Bryan I, Kenneth M, Niaman N, Aroop P, Melissa P. Reducing time to admission in emergency department patients: a cross-functional quality improvement project. BMJ Open Qual. 2022;11(3):e001987. 10.1136/bmjoq-2022-001987 PMC9486293 36122996

