# Supplementary material for: Predicting ICU transfer for high-risk patients upon medical admission via the medical intensive care prediction score (MICAPS)
Source: BMC Emerg Med. 2026 Jan 8;26:42. doi: 10.1186/s12873-025-01448-w (PMC12870378; doi:10.1186/s12873-025-01448-w)

**Supplementary Table 1:** Laboratory parameters among those admitted vs. those not admitted to ICU.

| **Lab Parameter** | **ICU Admission** | **Number of patients** | **Mean ± S.D./Median (IQR)** | **P-Value** |
| --- | --- | --- | --- | --- |
| **WBC** | No | 10778 | 8.7 (6.6-11.9) | <0.001 |
|  | Yes | 908 | 11.1 (7.9-15.6) |  |
| **Hgb** | No | 10779 | 12.3 ± 2.6 | 0.34 |
|  | Yes | 908 | 12.2 ± 3.1 |  |
| **Platelet** | No | 10578 | 263 (208-327) | <0.001 |
|  | Yes | 906 | 248.5 (181-325) |  |
| **Urea** | No | 10754 | 4.6 (3.3-7.4) | <0.001 |
|  | Yes | 908 | 5.7 (4-98) |  |
| **Creatinine** | No | 10757 | 78 (61-106) | <0.001 |
|  | Yes | 906 | 69 (92-143) |  |
| **Sodium** | No | 10663 | 137.3 ± 4.9 | 0.35 |
|  | Yes | 908 | 137.1± 6.1 |  |
| **Potassium** | No | 10557 | 4.1± 0.6 | 0.48 |
|  | Yes | 907 | 4.1 ± 0.8 |  |
| **Bicarbonate** | No | 10659 | 24.7 ± 4.1 | <0.001 |
|  | Yes | 907 | 22.4 ± 5.8 |  |
| **POC Glucose** | No | 6794 | 7.5 (5.7-11) | 0.92 |
|  | Yes | 830 | 7.4 (5.9-10.9) |  |
| **Lactic acid** | No | 7765 | 1.5 (1.1-2.1) | <0.001 |
|  | Yes | 767 | 2.2 (1.4-4) |  |
| **Bilirubin** | No | 9268 | 8 (5-12) | 0.005 |
|  | Yes | 864 | 9 (6-15.8) |  |
| **AST** | No | 9234 | 20 (16-30) | <0.001 |
|  | Yes | 897 | 27 (18-48) |  |
| **ALT** | No | 9472 | 19 (13-32) | <0.001 |
|  | Yes | 899 | 24.6 )16-45) |  |
| **CRP** | No | 8051 | 22.2 (5-84.4) | 0.002 |
|  | Yes | 808 | 25.8 (6-97.8) |  |
| **Procalcitonin** | No | 2798 | 0.2 (0.09-0.87) | <0.001 |
|  | Yes | 724 | 0.43 (0.13-2.28) |  |

**Supplementary Figure 1:** Distribution of ICU Admission Risk Prediction Score


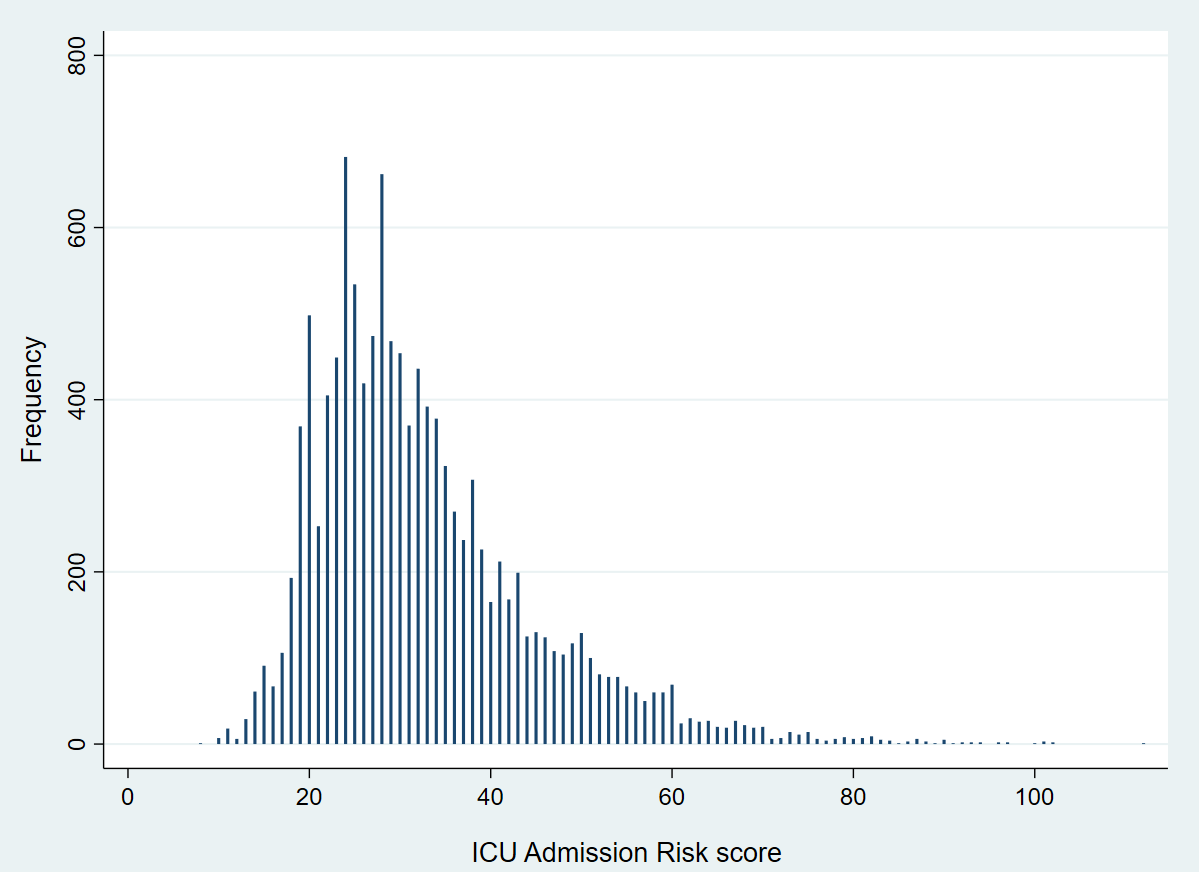


**Supplementary** **Figure 2:** Box plot depict distribution of weighted risk scores across ICU Admitted and non-ICU admitted cases


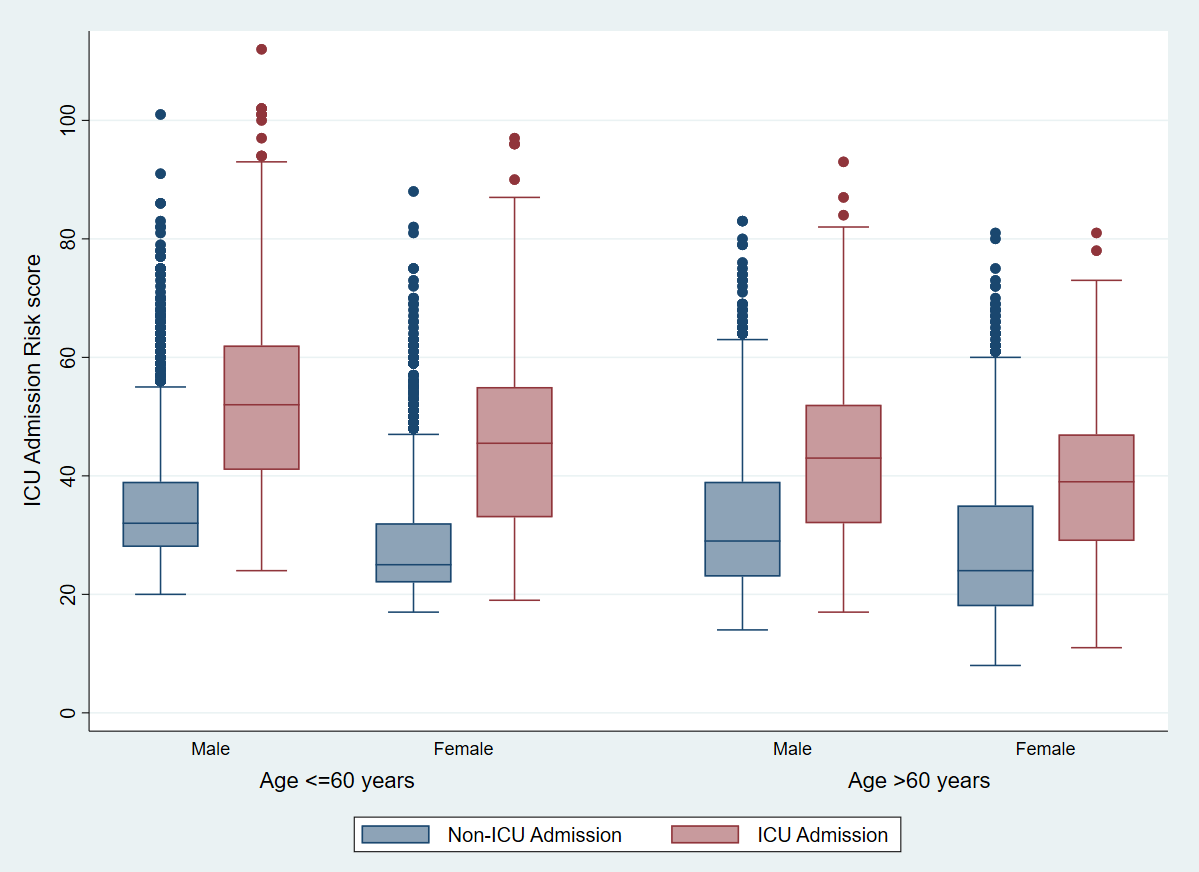


**Supplementary Figure 3:** Predictive accuracy evaluation of multivariate logistic regression model while excluding laboratory parameters using ROC curve indices.


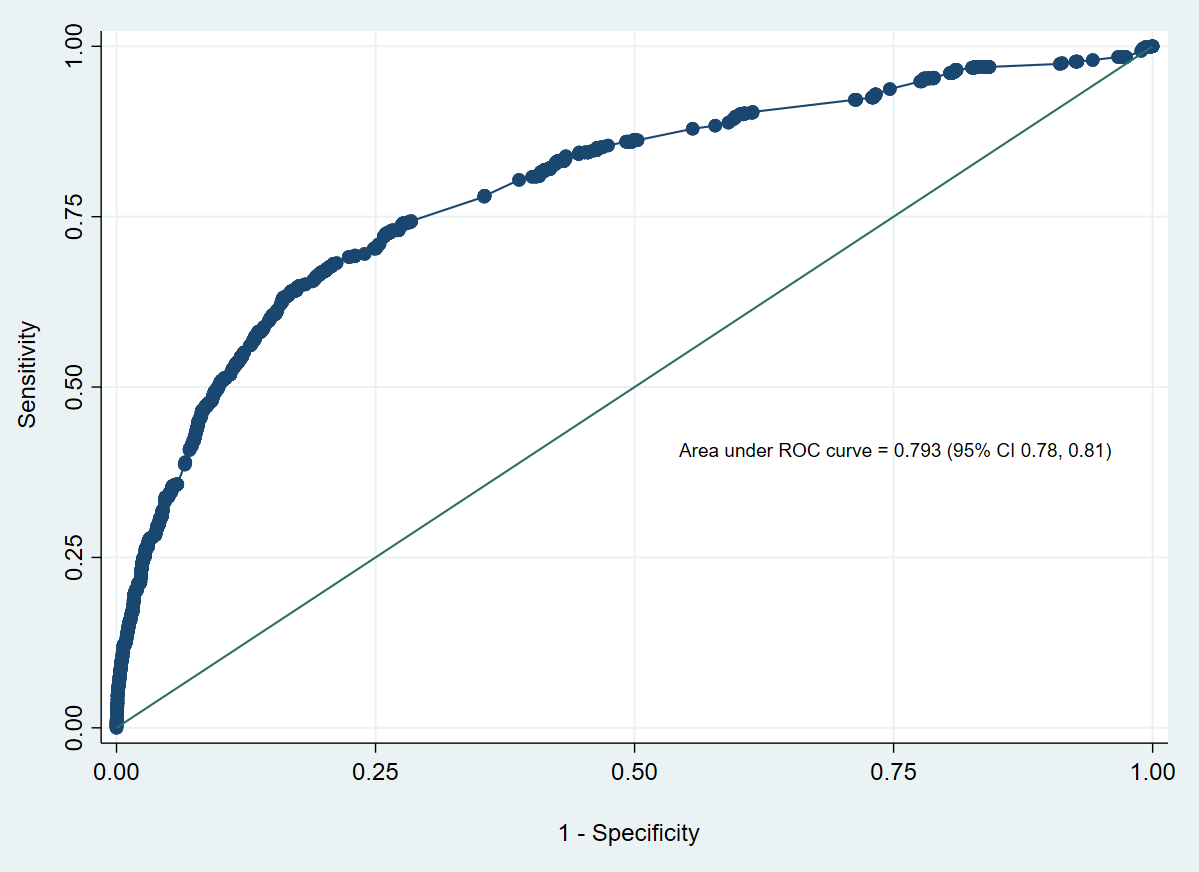

Supplement: Supplementary file 1 — Supplementary Material 1 [file 12873_2025_1448_MOESM1_ESM.docx]
